# Male Circumcision Utilising an Ultrasonic Dissection Scalpel in an Adult

**DOI:** 10.7759/cureus.79273

**Published:** 2025-02-19

**Authors:** Sarah K Whitehouse, Christian Beardsley

**Affiliations:** 1 General Surgery, Cairns Hospital, Cairns, AUS

**Keywords:** circumcision safety, male circumcision, ultrasonic device, ultrasonic instrumentation, ultrasonic surgical scalpel

## Abstract

A man in his 70s presented to surgical outpatients for phimosis and recurrent balanitis after previously receiving topical steroid treatment and a dorsal slit. He was medically comorbid including atrial fibrillation for which he was anticoagulated. He underwent a circumcision with an ultrasonic dissection scalpel (UDS). He recovered unremarkably and was able to recommence his anticoagulation on day 1 postoperatively. With the increasing age and comorbidities of many patients, this novel technique may be utilised to reduce operative time, postoperative bleeding risk and penile oedema.

## Introduction

Circumcision is one of the most performed operations in the world. Its indications can be varied, including cultural or religious traditions, medical necessity, personal hygiene or preventative health [1]. The reduction of urinary tract infections, sexually transmitted infections and penile cancer is well documented. The reduction of HIV rates is so pronounced that the World Health Organisation has funded a male circumcision program [1-5].

Due to its prevalence on an operative schedule, there is also significant debate about the best way to perform circumcision. Standard treatment around much of the world is the scalpel technique. Electrocautery, commonly referred to as diathermy, dissection of the layers of the foreskin, can cause damage to the surrounding tissue as its mechanism of action is to send an active current through the tissue to vaporise the tissue water until tissue reaches boiling point and tissue transection occurs [6]. Complications from monopolar diathermy documented in the literature include penile ablation, necrosis, gangrene and burns [7-8]. Altokhais completed a literature review, which recommended bipolar diathermy as safe if under the following conditions: small electrode tips, minimum energy settings and minimal application to tissues [9]. However, both options still open the surgeon to the significant risk of postoperative bleeding. Bleeding remains the second most common complication of circumcision using current techniques [1].

Other tools such as a myriad of clamps are on the market with the aim to improve practicality, decrease complication rates and be more affordable to patients [10]. They can reduce surgical time but have higher rates of excessive foreskin post-procedure than other techniques [10]. They usually are performed in an outpatient procedure setting with the practitioner using the clamp to apply pressure to the foreskin and aid in its removal. 

Ultrasonic dissection scalpels (UDS) utilise low-frequency ultrasonic sound waves with jaw pressure to simultaneously cut and coagulate tissue [6]. The technique uses lower temperatures and suffers less lateral spread of damage into the surrounding tissues [6]. Whilst widely used in open and laparoscopic surgery, it is still novel to utilise UDS in circumcision. UDS are expensive pieces of equipment that require an operating room with appropriate technology to be utilised. Animal studies have shown a significantly reduced operative time and decreased blood loss with fewer postoperative complications when compared to standard practice [11]. One case-control study in paediatrics presenting with phimosis showed technical success in all patients, no requirement for other instruments, a shorter operative time and no complications [12].

## Case presentation

A man in his 70s presented for surgical outpatient review for the treatment of chronic phimosis and balanitis. His issue was with foreskin retraction, making it difficult for him to maintain appropriate hygiene, had increased since a stroke five years prior to the review and progression of idiopathic tremor. He had previously undergone a dorsal slit, and his primary care physician had started topical steroid treatment, which had not improved his ability to retract his foreskin. 

His other significant medical history included ischemic heart disease with a previous coronary artery bypass graft, atrial fibrillation on DOAC and insulin-dependent type 2 diabetes mellitus with very poor glycaemic control. His multiple medical issues and general frailty were noted. 

On examination, he had findings consistent with phimosis and lichen sclerosis. Preoperatively, the patient withheld his DOAC for three days. 

The procedure was done under penile local anaesthetic block and light sedation. A handheld Harmonic Focus + Shears (Ethicon, Johnson and Johnson) was used to perform a circumcision of the foreskin. Using a technique with similar principles to the scalpel technique, the UDS was set at the standard levels of 5 and 3. With protection of the urethra, a dorsal incision and resection of the outer layer of the prepuce were made utilising coagulation at level 5. The inner layer of the prepuce was completed at level 3. There was no significant bleeding that occurred. 

3.0 Vicryl Rapide suture (Ethicon, Johnson and Johnson) was chosen to reinforce the coronal sulcus due to its ability to rapidly absorb and therefore not require removal. Chloramphenicol ointment was placed over the top as the dressing. A complete theatre time, including administration, preparation, local anaesthetic block and operation, was 45 minutes. 

**Figure 1:**
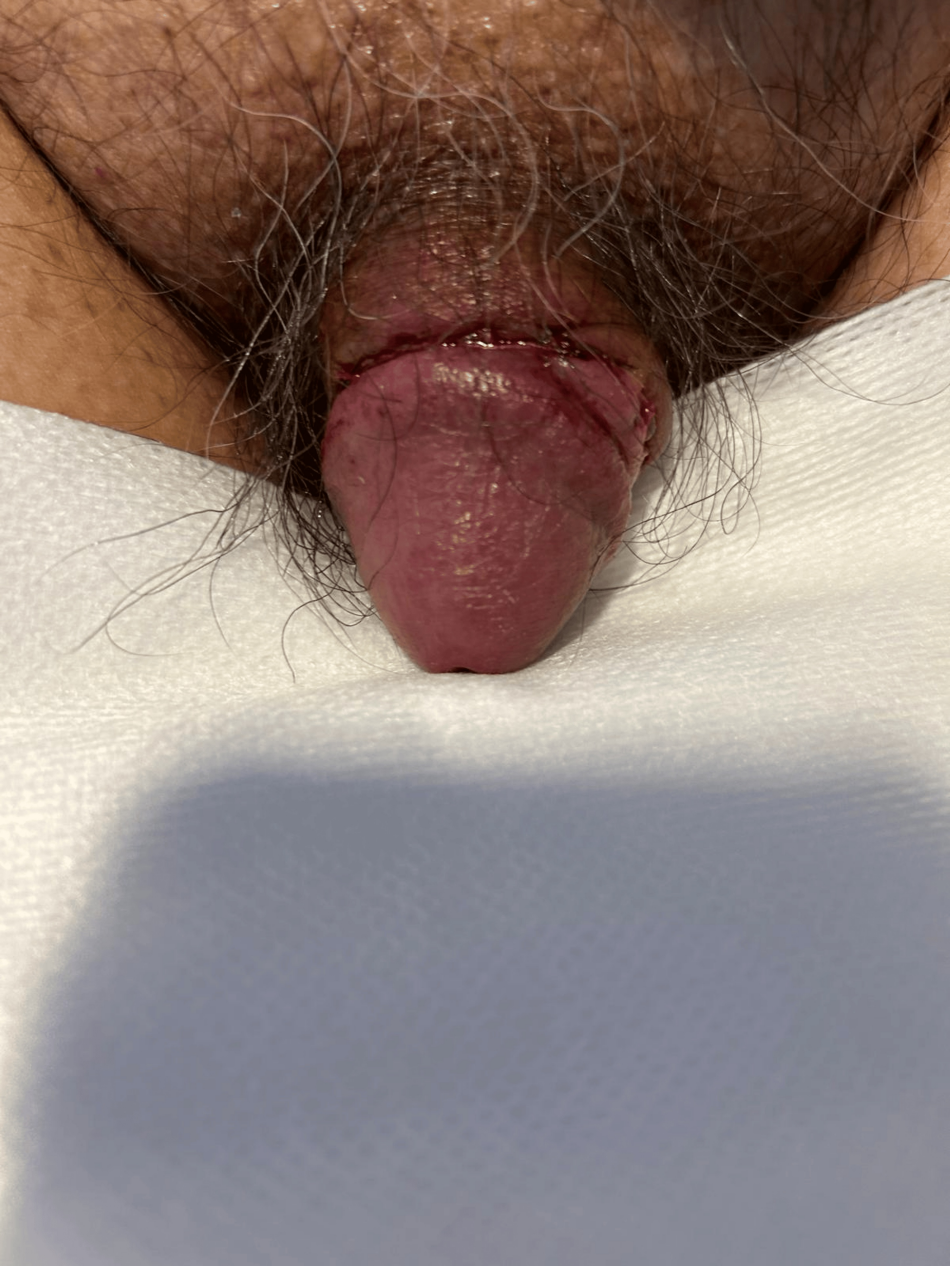
Day 1 postoperative image of penis post circumcision using an ultrasonic dissecting scalpel. There is no postoperative bleeding or oedema of the penis.

The patient remained in the hospital overnight as he resided 1.5 hours' drive away and his procedure was late in the day. He was reviewed in the morning and had no signs of bleeding or oedema of the penis. He was discharged on day 1 post-procedure and restarted his anticoagulation that day. Postoperative images were taken with the patient's consent (see Figure 1).

His treating surgeon called three days later for follow-up, and there was no bleeding on resumption of his anticoagulation, pain or oedema in his penis. Due to his significant geographical distance from the hospital, he was followed up further by his primary care physician.

## Discussion

This is the first documented use of UDS for circumcision in an adult patient in the literature. We used the same technique as described by the only case series published [12].

We chose to utilise the UDS for this procedure due to its well-documented reduction in bleeding risk [13-16]. The superior haemostasis gained from the UDS allowed us to start his anticoagulation sooner postoperatively and with decreased concern for postoperative bleeding and re-operation. This was of increased importance as this patient was frail and medically vulnerable and lived in a very rural location. 

As populations age, it is likely that the frequency of circumcision in older and more medically comorbid patients will increase. By utilising this technique, we can minimise their time off anticoagulation, decreasing their risks of thrombosis and its sequelae. Other complications such as infection, severe oedema and damage to the surrounding tissue were not seen in our case or the two other published papers in the literature [11-12]. It also allows for short operative time and the use of only one instrument to complete. We acknowledge that we can only confirm short-term complications were not experienced. Further studies with longer-term follow-up may help to determine the UDS efficacy compared to other, more common options.

## Conclusions

This case report shows the safety of utilising the UDS in circumcision to improve risks of penile oedema and minimise postoperative bleeding in the short-term postoperative period. This is consistent with previous documented case series in children. However, further research should be undertaken to determine its comparison to other studies. 

## References

[1] Gologram M, Margolin R, Lomiguen CM (2022). Need for increased awareness of international male circumcision variations and associated complications: a contemporary review. Cureus.

[2] Hargreave T (2010). Male circumcision: towards a World Health Organisation normative practice in resource limited settings. Asian J Androl.

[3] Auvert B, Sobngwi-Tambekou J, Cutler E, Nieuwoudt M, Lissouba P, Puren A, Taljaard D (2009). Effect of male circumcision on the prevalence of high-risk human papillomavirus in young men: results of a randomized controlled trial conducted in Orange Farm, South Africa. J Infect Dis.

[4] Bailey RC, Moses S, Parker CB (2007). Male circumcision for HIV prevention in young men in Kisumu, Kenya: a randomised controlled trial. Lancet.

[5] Gray RH, Kigozi G, Serwadda D (2007). Male circumcision for HIV prevention in men in Rakai, Uganda: a randomised trial. Lancet.

[6] Stefan H (1994). Reconstruction of the penis after necrosis due to circumcision burn. Eur J Pediatr Surg.

[7] McCarus SD, Parnell LK (2019). The origin and evolution of the HARMONIC® scalpel. Surg Technol Int.

[8] Uzun G, Ozdemir Y, Eroglu M, Mutluoglu M (2012). Electrocautery-induced gangrene of the glans penis in a child following circumcision. BMJ Case Rep.

[9] Altokhais TI (2017). Electrosurgery use in circumcision in children: is it safe?. Urol Ann.

[10] Tuncer AA, Erten EE (2017). Examination of short and long term complications of thermocautery, plastic clamping, and surgical circumcision techniques. Pak J Med Sci.

[11] Peng M, Meng Z, Yang ZH, Wang XH (2013). The ultrasonic harmonic scalpel for circumcision: experimental evaluation using dogs. Asian J Androl.

[12] Fette A, Schleef J, Haberlik A, Seebacher U (2000). Circumcision in paediatric surgery using an ultrasound dissection scalpel. Technol Health Care.

[13] Dedivitis RA, de Matos LL, Castro MA, Petrarolha SM, Kowalski LP (2024). Neck dissection with harmonic instruments and electrocautery: a systematic review. Int Arch Otorhinolaryngol.

[14] Qaiser MU, Nazir A, Khan MS, Butt HK, Anwar M (2021). Comparison of ultrasonic dissection and suture ligation for mesoappendix in laparoscopic appendectomy. Cureus.

[15] Kloosterman R, Wright GW, Salvo-Halloran EM (2023). An umbrella review of the surgical performance of Harmonic ultrasonic devices and impact on patient outcomes. BMC Surg.

[16] Wiatrak BJ, Willging JP (2002). Harmonic scalpel for tonsillectomy. Laryngoscope.

